# Capital stock, energy, and innovation-related aspects as drivers of environmental quality in high-tech investing economies

**DOI:** 10.1007/s11356-022-24148-5

**Published:** 2022-12-24

**Authors:** Ali Celik, Andrew Adewale Alola

**Affiliations:** 1 Department of International Trade and Finance, Istanbul Gelisim University, Istanbul, Turkey; 2 Centre for Research on Digitalization and Sustainability (CREDS), Inland Norway University of Applied Science, 2418 Elverum, Norway; 3 Department of Economics and Finance, South Ural State University, Chelyabinsk, Russia

**Keywords:** Carbon policy, Innovation, Sustainable technology, R&D, Developed economies, Empirical analysis

## Abstract

By looking at the technological advancement and climate change mitigation plan of the advanced economies, the current study examines the role of sustainable development aspects such as innovations, high technology export, labor productivity, capital stock, research and development (R&D), information and communication technology (ICT), capital stock, and energy use in mitigating environmental degradation for the selected panel of countries with the most investment in technology (China, Denmark, Finland, France, Israel, Korea, Hong Kong, Germany, Japan, Netherlands, Singapore, Sweden, United Kingdom, and United States) over the period 2000–2018. Foremost, the pooled ordinary least square (POLS) and random-effects (RE) generalized least squares (GLS) approaches provided additional interesting inferences. As such, the POLS result revealed that only capital stock in the panel countries shows a desirable environmental effect. At the same time, labor productivity, innovation, R&D, ICT, and energy further hamper ecological quality in the examined panel countries. Similarly, the GLS result largely affirms the POLS results, with only the capital stock among the explanatory variables showing evidence of emission mitigation effect in the panel. Additionally, the panel Granger causality result illustrates evidence of unidirectional causality only innovation, ICT, and capital stock to environmental degradation.

## Introduction

While it has been widely acknowledged that the burning of conventional energy resources is largely responsible for the challenges associated with global warming, the seemingly favorable effect of technological innovations on the environment has increasingly been investigated. To attain the target of the Kyoto Protocol of the United Nations Framework Convention on Climate Change and the Global goal vis-a-vis the Sustainable Development Goals (SGDs) of the United Nations Development Program (UNDP), the pathway to achieving climate mitigation has been mostly designed along with green technology policy. In this respect, Du et al. (2019) alluded to the importance of renewable or cleaner technologies, research and development (R&D), and the diffusion of green technological innovation (such as the improved concentrated solar power (CSP)) in driving down the agent of global warming such as the greenhouse gas (GHG) emissions. As the worldwide debate on climate change persists, isotopic techniques and carbon technologies, such as carbon capture and storage (CCS), carbon capture and utilization (CCU), and carbon capture and sequestration (CCS), are being significantly intensified to complement energy technologies.

Moreover, other silent forms of innovations such as patent registration are potentially linked with carbon dioxide (CO_2_) emission (Dinda 2018; Ganda 2019). In terms of the role of digital technologies, the World Economic Forum (WEF) highlighted the potential to achieve a 15% decline in GHG emissions by 2030 (World Economic Forum 2019). Additionally, to provide climate mitigation prospects, the development of high technologies significantly offers diverse opportunities, thus driving the related aspects of technological investments. For instance, the recent report of PricewaterhouseCoopers indicates that the mobility and transportation sectors have mostly benefited from green technology investment, followed by land use and agriculture and the energy sector (PricewaterhouseCoopers 2020). Furthermore, the report further hinted that e-scooter and bike platforms which are the common types of micro-mobility in addition to other varieties of transport innovations are now hugely invested across major economies of the world (giving it a compound annual growth rate of 151%).

By considering the motivations mentioned above, the objective of the current study is drawn from the notion of examining the role of innovation-related factors on GHG vis-a-vis carbon emissions. While experimenting from the case of the selected developed economies that have the most investment in high technology (China, Denmark, Finland, France, Israel, Korea, Hong Kong, Germany, Japan, Netherlands, Singapore, Sweden, United Kingdom, and the United States), the specific hypothesis is being investigated in line with the objective mentioned above. The novelty of the study is not only about the case in consideration. The study offers an extensive examination given that the hypotheses are derived from the roles of patent applications (i) R&D, (ii) high technology, (iii) information and communication technology (ICT), (iv) capital stock (CS), and (vi) energy on carbon dioxide (CO_2_) emission for the panel countries as mentioned above over the covering 2000–2018. By ensuring a robust investigation, additional variables (labor productivity) were incorporated.

We arrange the remaining sections of the study accordingly. Several relevant pieces of literature are discussed in “Related literature” while presenting the data and preliminary tests in “Theoretical literature”. In “Data and preliminary tests”, we carry out the co-integration and Granger causality analyses while discussing the results in “Model and preliminary tests”. The last section, “Empirical analysis and results”, is reserved for the conclusion and policy insight of the study.

## Related literature

Considering that the current study is centered on the case of selected developed high-tech investing economies, a related topic and approach is detailed in the recent work of Erdoğan et al. (2020). Specifically, Erdoğan et al. (2020) examined the role of innovation in the carbon emission drive (especially the sectoral carbon emissions) for 14 of the G-20 countries covering the period of 1991–2017. While the study ruled out the validity of the Environmental Kuznets curve (EKC) hypothesis for the panel examination, it further found that the role of innovation in the carbon emissions across the sectors (such as the energy sector and transport sector) is not statistically significant in the long run. However, the short-run observation poses an interesting perspective. Thus, in the short run, innovation yields improvement in environmental quality (mitigate carbon emission) through the industrial sector while causing the opposite effect in the construction sector. By examining the case of Canada (one of the countries being considered in the current study), Jordaan et al. (2017) informed that the country’s energy transition effort through renewable portfolio measures, high carbon fuel phase-out, and other clean technologies is significantly yielding emission mitigation goals.

In the recent study by Ganda (2019), the dimensions to carbon mitigation from the perspectives of the patent application, innovation, technological investment, and research and development were examined for the case of the Organization for Economic Co-operation and Development (OECD) economies. The study offers interesting results by implementing the system-generalized method of moments (GMM) technique from 2000 to 2014. In the study, the result established that two indicators (spending on R&D and renewable energy utilization) were found to prevent the outrush of carbon emission on a significant term in the panel model. However, the number of triadic patent families is revealed to trigger more environmental setbacks. In contrast, the number of researchers marks a potential push in causing environmental damage (but the impact is statistically not significant). The general observation from the entire result is that technology investments and innovation possess the ability to mitigate carbon emissions, especially by showing the different effects on carbon emissions in the examined OECD countries.

Similarly, a related inference from the nexus of carbon emission and green technology innovations was offered in the work of Du et al. (2019). Based on the panel analysis of selected 71 economies covering from 1996 to 2012, Du et al. (2019) employed the panel fixed effect model to examine the impact of green patent counts, energy consumption, trade openness, and the validity of the environmental Kuznets curve. Specifically, the study found that green technology innovations (as proxied by green patent count) reduce carbon emission in only some of the different economic levels. The desiring effect of green technology innovations on carbon emission is only significant in high-income level economies. Moreover, the study established the EKC hypothesis in the framework of environmental degradation and green technological innovation nexus. These highlighted studies are close reflections of the revealing insight of the study of Cheng et al. (2021) that also found an insignificant relationship between carbon emission and patent development.

Additionally, the dynamics of carbon emissions from the perspectives of both technological innovation and gains in efficiency for China were examined by Zhang et al. (2016). By proposing the relevance of the non-radial global Malmquist carbon emission performance index (NGMCPI) to overcome inherent challenges in previous estimation approaches, Zhang et al. (2016) examined the dynamic carbon emission performance (CEP) across 38 industrial sectors in China covering the period 1990–2012. The study found that the NGMCPI is capable of overcoming the challenge of decoupling CEP from radial efficiency measures and the infeasibility issue associated with the estimation process. Specifically, the NGMCPI is decomposed into the low-carbon catch-up (called the efficiency change) and the innovation effects (called the technological change) indexes. Thus, the study found that the low-carbon catch-up drove the dynamic (CEP) during the 1990s, while innovation further complemented the trigger between 2000 and 2012.

Furthermore, the studies of Khan et al. (2020) and Shahbaz et al. (2020) considered the role of investment through public–private partnerships and innovations in technological advancement in mitigating emissions in China. By employing the bootstrapping autoregressive distributed lag (ARDL) technique, Shahbaz et al. (2020) found that technological innovations and public–private partnerships respectively mitigate and induce carbon emissions while both validating the EKC hypothesis. Thus, the study offered a concrete policy for the Chinese government especially as the country continues to battle the challenge associated with GHG emission. Although Khan et al. (2020) implemented a different set of co-integration approaches (such as the Maki co-integration, fully modified ordinary least square, dynamic ordinary least square, and canonical co-integration regression) along with frequency domain causality test, the observation is similar to that of Shahbaz et al. (2020). Specifically, Khan et al. (2020) found that technological innovation along with exports and renewable energy utilization is a significant measure toward the carbon emission mitigation approach in China. Additionally, the study found that public–private partnerships, import, and economic growth spur environmental degradation through the outrush of carbon.

Moreover, there are several other recent and related studies that align with the framework of the current study (Adedoyin et al. 2020; Godil et al. 2021; Du et al. 2022; Sun et al. 2021; Abbasi et al. 2022; Chien, et al. 2022; Onifade and Alola 2022). For instance, Godil et al. (2021) employed the quantile autoregressive distributed lag (QARDL) approach for the dataset covering 1990–2018 to examine the roles of technology innovation, conventional and unconventional energy resources in transport carbon emission in China. Specifically, the study reveals that advancement in technological innovation and renewable energy resource utilization are significantly leading to a decline in carbon emission in the country’s transport sector. At the same time, economic growth (measured by GDP) causes a surge in CO_2_ emission in the sector. Similarly, Abbasi et al. (2022) examined Pakistan’s by deploying the dataset covering 1990Q1 to 2019Q4 and the newly developed dynamic ARDL. Importantly, the study shows that technological innovation reduces consumption and territory-based CO_2_ emissions, especially in the long run. At the same time, economic development, total energy utilization, and economic globalization largely spur consumption and territory-based CO_2_ emissions in the long run.

While the studies mentioned above clearly demonstrate a significant revelation, especially about the role of innovation aspects on environmental quality, the current study provides a more expansive coverage and depth of the literature. In essence, the current study does not only incorporate a spread of the innovation aspects in the same model, the focus of the study, i.e., the high-tech-investing economies, also makes the current study a unique endeavor.

### Theoretical literature

**Fig. 1:**
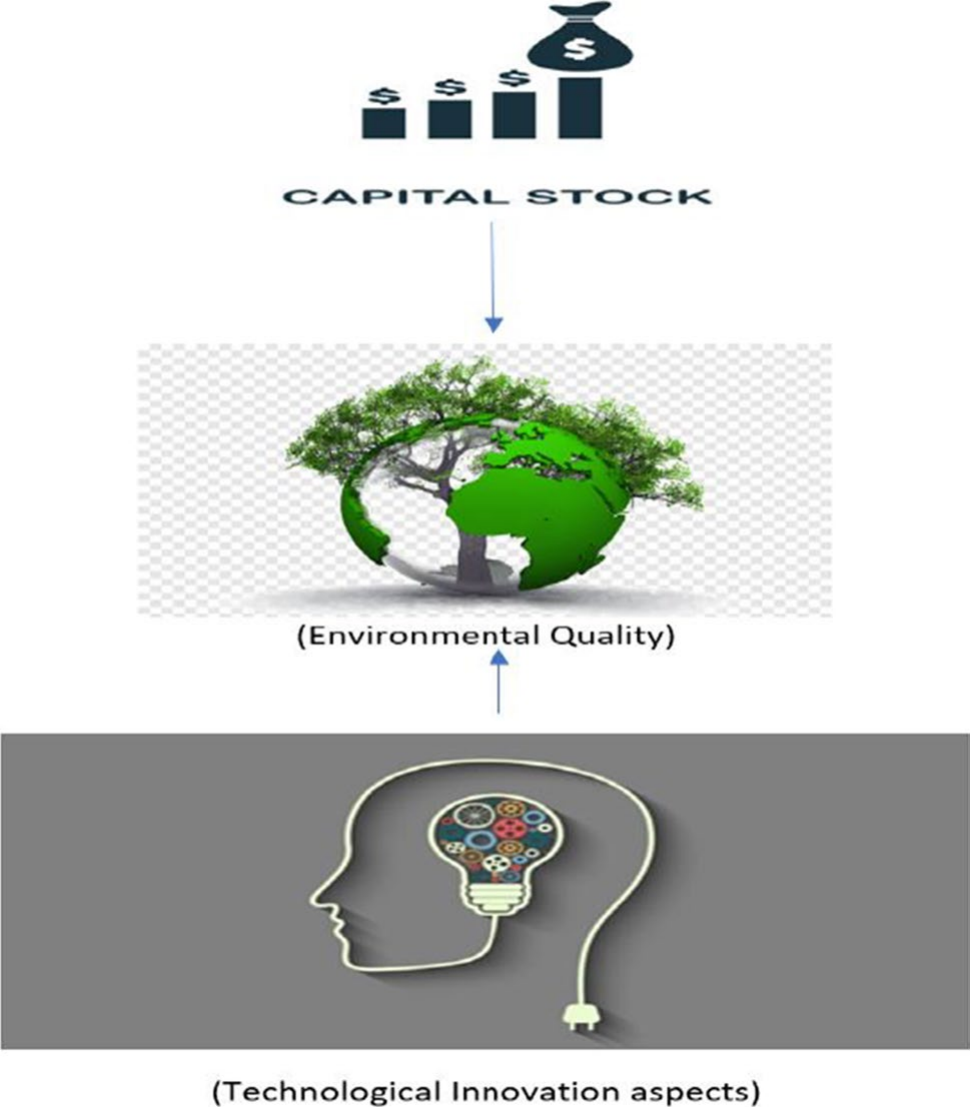
Conceptual illustration of the study

Reflecting on the proposition of the growth model, such as the work of Solow (1956) that is centered on the key drivers of economic growth, successive modifications have paved the way for the relevance of other growth factors. In addition, the increasing relevance of knowledge and technological change as essential resources for growth has yielded more evidence over time (Abramovitz 1956; Arrow 1971). Moreover, while looking at technological innovation from the perspective of patent (a proxy for innovation), as hinted by Griliches (1998), Dinda (2018) used the utility patent (UTPAT) to represent production technology and at the same time to understand the carbon emission effect. Considering that pollution is inherent in the production process, the pollution aspect is derived from the relationship between output production and technological improvement. Earlier, Ehrlich and Holdren (1971) and their similar study (Holdren and Ehrlich 1974) illustrated the impact (*I*) of affluence (denoted as *A* = economic growth), population (denoted as *P*), and technological advancement (denoted as *T*), i.e., IPAT which was later modeled stochastically in a subsequent study. Thus, given that pollution per unit output (*µ*) decreases with technological innovation, then pollution = $\frac{\mathrm{Output}(\mu )}{A}$, where 0 < *µ* < 1 and *A* is the technological innovation parameters. In the case of the current study, the task expects to establish the environmental effect of technological innovation aspects, labor productivity, and capital stock as captured in the illustrated Fig. 1.

## Data and preliminary tests

This study utilizes the annual data covering 2000 to 2018 for the most innovative countries^1^ as determined by the World Intellectual Property Organization (WIPO 2021) to examine the relationship between technological variables and carbon dioxide emissions. Modeling variables are illustrated in Table 1.

**Table 1:** Definition of variables

Variable	Code	Unit	Source
Carbon dioxide emissions	$${CO2}_{it}$$	Metric tons	Maddison
Labor productivity	$${LP}_{it}$$	Output per worker	Own calculated
Patent applications of residents	$${PA}_{it}$$	Number of patent	WIPO
Research and development expenditure (% of GDP)	$${RD}_{it}$$	Percent	World Bank
Information and communication technology goods exports (% of total goods exports)	$${ICT}_{it}$$	Percent	World Bank
Capital stock at current PPPs (in mil. 2017 US$)	$${CS}_{it}$$	Current purchasing power parities in million 2017 USA dollars	PWT10.0
Energy^2^	$${EN}_{it}$$	Index	UNCTAD

PWT: Penn World Table; UNCTAD: United Nations Conference on Trade and Development. All data were used with logarithmic transformation

^2^This category measures the availability, sustainability, and efficiency of power sources. For this reason, it is composed of the use of and access to energy, losses in distribution, and renewability of energy components and sources and includes the GDP generated by each unit of oil to further highlight the importance of optimal energy systems (UNCTAD)

### Model and preliminary tests

This study aims to examine the effect of technology parameters on carbon dioxide emissions. In this context, the mathematical and econometric model is given by the following:

1 $${lnCO}_{2}=f(lnLP, lnPA, lnRD, lnICT, lnCS, lnEN)$$

From Eq. (1), the form of econometrics model is further represented as

2 $${lnCO2}_{it}={\beta }_{0}+{\beta }_{1}\mathrm{ln}{LP}_{it}+{\beta }_{2}\mathrm{ln}{PA}_{it}+{\beta }_{3}ln{RD}_{it}+{\beta }_{4}{ICT}_{it}+{\beta }_{5}ln{CS}_{it}+{\beta }_{6}ln{EN}_{it}+{\mu }_{i}+{\mu }_{it}$$

with *i* denoting countries (1,2,3,…,*N*) and *t* denoting time (2000, 2001, 2002,…,*T*). The subscript *i*, therefore, states the cross-sectional dimension whereas *t* states the time series dimension. *Β*_0_ is constant term, *μ*_*i*_ is the unobservable individual-specific effect, and *u*_*it*_ is an idiosyncratic error term. ln displays the natural logarithmic. Additionally, since the econometric model has a full logarithmic form, the parameters should be interpreted by taking this into account. As seen in Table 2, this study is conducted using 266 observations. The natural logarithm of the variables is taken to eliminate scale differences and to calculate the slope coefficients. While the mean of *lnCO*_2_, *lnLP*, *lnPA*, and *lnRD* is calculated as 2.11, 11.28, 9.20, and 0.84, respectively, the mean of the *lnICT*, *lnCS*, and *lnEN* is calculated as 2.47, 15.5, and 3.54, respectively. The standard deviations of the variables are found as 0.36, 0.50, 2.42, and 0.45 for *lnCO*_2_, *lnLP*, *lnPA*, and *lnRD*, respectively, and 0.78, 1.41, and 0.06 for *lnICT*, *lnCS*, and *lnEN*, respectively. Additionally, the maximum and minimum values of the variables were estimated; the difference between the maximum and minimum values is substantially low due to the logarithmic transformation.

**Table 2:** Descriptive statistics

Variable	Obs	Mean	Std. Dev	Min	Max
$ln{CO2}_{it}$	266	2.11	0.36	0.97	3.02
$ln{LP}_{it}$	266	11.28	0.50	8.90	11.78
$ln{PA}_{it}$	266	9.20	2.42	3.93	14.15
$ln{RD}_{it}$	266	0.84	0.45	− 0.77	1.60
$ln{ICT}_{it}$	266	2.47	0.78	0.84	4.02
$ln{CS}_{it}$	266	15.51	1.41	13.50	18.35
$ln{EN}_{it}$	266	3.54	0.06	3.30	3.68

Natural logarithms of all variables were taken to eliminate scale differences

A close relationship in all or at least two of the explanatory variables is called multi-collinearity. Ordinary least square (OLS) estimators are best linear unbiased estimator (BLUE). Specifically, in the case of multi-collinearity, the OLS estimates are unbiased, but the estimates may diverge from their true values as the variances become larger. For this reason, it is aimed to avoid this problem by employing some methods. The variance inflation factor (VIF) criterion is used to determine whether there is a multi-collinearity problem. If VIF < 5, there is no multi-collinearity problem, if 5 < VIF < 10, there is a moderate multi-collinearity problem, and if VIF > 10, there is a high multi-collinearity problem. Table 3 shows that the results of the VIF criteria are determined to be less than 5 for both the independent variables and the mean. This result indicates that there is no multi-collinearity problem in the model.

**Table 3:** Variance inflation factor (VIF)

Variable	VIF	1/VIF
$ln{LP}_{it}$	2.60	0.38
$ln{PA}_{it}$	1.88	0.53
$ln{RD}_{it}$	1.80	0.55
$ln{CS}_{it}$	1.63	0.61
$ln{EN}_{it}$	1.40	0.71
$ln{ICT}_{it}$	1.33	0.75
Mean VIF	1.77	

Table 4 shows the individual effect and time effect results. Individual effects and time effects are often used to test for individual or time heterogeneity that is not observed in panel data models (Arellano 2003; Wooldridge 2010; Baltagi 2006; Hsiao 2014). There are various tests in the literature to test the presence of fixed effects in one or multi-dimensional panel data models. Most tests focus on static panel models. In this study, the hypothesis is separately examined for F, LR, and LM tests.

**Table 4:** The hypothesis for F, LR, LM, and Hausman tests

Tests	Null hypothesis	Alternative hypothesis	Decision phase
Hypothesis for F test	${H}_{0}: {\mu }_{i}={\lambda }_{t}=0$	${H}_{1}: {\mu }_{i} or {\lambda }_{t}\ne 0$	Is the model classic or not?
	${H}_{0}: {\mu }_{i}=0$	${H}_{1}: {\mu }_{i}\ne 0$	Does the model have individual effects or not?
	${H}_{0}: {\lambda }_{t}=0$	${H}_{1}: {\lambda }_{t}\ne 0$	Does the model have time effects or not?
Hypothesis for LR test	${H}_{0}: {\sigma }_{\mu }={\sigma }_{\lambda }=0$	${H}_{1}: {\sigma }_{\mu }or {\sigma }_{\lambda }\ne 0$	Is the model classic or not?
	${H}_{0}: {\sigma }_{\mu }=0$	${H}_{1}: {\sigma }_{\mu }\ne 0$	Does the model have time effects or not?
	${H}_{0}: {\sigma }_{\lambda }=0$	${H}_{1}: {\sigma }_{\lambda }\ne 0$	Time effects model or not?
Hypothesis for LM test	${H}_{0}: {\sigma }_{\mu }^{2}={\sigma }_{\lambda }^{2}=0$	${H}_{1}: {\sigma }_{\mu }^{2}or {\sigma }_{\lambda }^{2}\ne 0$	Is the model classic or not?
	${H}_{0}: {\sigma }_{\mu }^{2}=0$	${H}_{1}: {\sigma }_{\mu }^{2}\ne 0$	Does the model have individual effects or not?
	${H}_{0}: {\sigma }_{\mu }^{2}\ne 0$	${H}_{1}: {\sigma }_{\lambda }^{2}\ne 0$	Does the model have time effects or not?
Hypothesis for Hausman test	FE is consistent whereas RE is efficient	FE is consistent whereas RE is inconsistent	Which model FE or RE?

FE and RE denote fixed effects and random effects, respectively

The null hypothesis of these tests shows that there is no individual effect, time effect or both, whereas the alternative hypothesis implies that there exists individual effect, time effect, or both, respectively. In this context, we test the following models to determine the correct model:

3 $${lnCO2}_{it}={\beta }_{0}+{\beta }_{1}ln{LP}_{it}+{\beta }_{2}ln{PA}_{it}+{\beta }_{3}ln{RD}_{it}+{\beta }_{4}{ICT}_{it}+{\beta }_{5}ln{CS}_{it}+{\beta }_{6}ln{EN}_{it}+{u}_{it}$$

4 $${lnCO2}_{it}={\beta }_{0}+{\beta }_{1}ln{LP}_{it}+{\beta }_{2}ln{PA}_{it}+{\beta }_{3}ln{RD}_{it}+{\beta }_{4}{ICT}_{it}+{\beta }_{5}ln{CS}_{it}+{\beta }_{6}ln{EN}_{it}+{u}_{i}+{u}_{it}$$

5 $${lnCO2}_{it}={\beta }_{0}+{\beta }_{1}ln{LP}_{it}+{\beta }_{2}ln{PA}_{it}+{\beta }_{3}ln{RD}_{it}+{\beta }_{4}{ICT}_{it}+{\beta }_{5}ln{CS}_{it}+{\beta }_{6}ln{EN}_{it}+{\lambda }_{t}+{u}_{it}$$

6 $${lnCO2}_{it}={\beta }_{0}+{\beta }_{1}ln{LP}_{it}+{\beta }_{2}ln{PA}_{it}+{\beta }_{3}ln{RD}_{it}+{\beta }_{4}{ICT}_{it}+{\beta }_{5}ln{CS}_{it}+{\beta }_{6}ln{EN}_{it}+{u}_{i}+{\lambda }_{t}+{u}_{it}$$

with *i* denoting countries and *t* denoting time for this study. *Β*_0_ is constant term, *μ*_*i*_ is the unobservable individual-specific effect, *λ*_*t*_ is the unobservable time-specific effect, and *u*_*it*_ is an idiosyncratic error term. Equation (3) shows the classical model in which there is neither an individual effect nor a time effect. In the presence of such a model, the pooled least squares estimation results can be relied upon. While Eq. (4) expresses the one-way error component model with the individual effects, Eq. (5) shows the one-way error component model with time effects model. Equation (6) shows the two-way error component model in which both effects exist together. If the pooled least squares estimation method is used in models with individual or time effects, biased results may occur. Against this situation, the use of fixed-effects and random-effects models is recommended. As seen in Table 4, it has been shown that there is no time effect in the F, LR (Likelihood ratio), and LM (Lagrange multiplier by Breusch and Pagan 1980) tests. In other words, the null hypothesis is accepted based on three tests. However, the alternative hypothesis that there is an individual effect is accepted.

Accordingly, Eq. (2) is our final model for analysis. The econometric model (Eq. (2)) can be constructed under the presence of the individual effect. In addition, Hausman (1978) suggests comparing ${\widehat{\beta }}_{\mathrm{GLS}}$ and${\widetilde{\beta }}_{\mathrm{Within}}$, both of which are consistent under the null hypothesis ${H}_{0} :E({u}_{it}/{X}_{it}) =0,$ but which will have different probability limits if ${H}_{0}$ is not true. In fact, ${\widetilde{\beta }}_{\mathrm{Within}}$ is consistent whether ${H}_{0}$ is true or not, while ${\widehat{\beta }}_{\mathrm{GLS}}$ is BLUE (best linear unbiased estimator) consistent and asymptotically efficient under${H}_{0}$, but is inconsistent when ${H}_{0}$ is false (Baltagi 2005: 67). On the other hand, the results of the Robust Hausman (rhausman) test used to determine whether the individual effect is related to the independent variable allow the use of the random-effects model, which can be used when the individual effect is not related to the independent variable. Accordingly, the Hausman test arising from the results of the time and individual effect is displayed in Table 5.

**Table 5:** Results of F, LR, LM, and Hausman test

Tests	Individual effect		Time effect	
	Test stat	Prob	Test stat	Prob
F	138.21	(0.000)	0.46	(0.972)
LR	453.1	(0.000)	0.00	(1.000)
LM	1624.25	(0.000)	0.00	(1.000)
Hausman test				
Robust Hausman	Prob > chi^2^ = 0.84			

^*^5% significance level

Table 6 illustrates mainly the results of deviations from assumption. The normality test results for random effects initially demonstrate that the null hypothesis is accepted within the 5% significance level. In other words, the individual effect error component and the residual error are normally distributed. By considering the heteroscedasticity, all models are compared with the Snedecor F table for Levene et al. heteroscedasticity tests. Accordingly, the null hypothesis stating that there is no heteroscedasticity is rejected. This result proves the existence of heteroscedasticity. Moreover, the Durbin Watson (DW) and LB autocorrelation test results are smaller than critical values (it is accepted as ‘‘2’’). Accordingly, these results denote there is first-order autocorrelation. The cross-sectional dependence is also found for this study. Statistically, there are deviations from the assumption; thus, the robust estimators should be used (see Table 6). The robust estimators help to produce effective results against deviations from assumptions such as heteroscedasticity, autocorrelation, and cross-sectional dependence as respectively proposed by Brown and Forsythe (1974), Bhargava et al. (1982), and Pesaran (2004).

**Table 6:** Testing for normality, heteroscedasticity, autocorrelation, and cross-sectional dependence

		Prob
Normality test	${\mu }_{i}$	0.217
	${u}_{it}$	0.607
Brown, and Forsythe test for heteroscedasticity	${W}_{0}$= 7.955	0.000^*^
	${W}_{50}$= 3.929	0.000^*^
	${W}_{10}$= 6.980	0.000^*^
DW test proposed by Bhargava et al. (1982) and LBI test proposed by Baltagi-Wu for autocorrelation		0.385
		0.608
Pesaran’s test of cross-sectional independence	6.531	0.000^*^
Friedman’s (1937) test of cross-sectional independence	55.624	0.000^*^

^*^5% significance level. The autocorrelation test results are compared with the critical value determined as 2

### Empirical analysis and results

To estimate of the coefficient relationship between the environmental variable and the set of explanatory variables, we adopt the Driscoll-Kraay standard error robust estimator. The consideration of this approach is because the Driscoll-Kraay standard error robust estimator is highly effective in estimating models where deviations from these three assumptions occur simultaneously. F and Wald test results show that the equations are statistically significant, respectively. As seen in Table 7, we apply two different estimators based on Driscoll-Kraay standard error. Panel (a) provides the pooled least squares (POLS) results, while panel (b) presents the random effects (RE) generalized least squares (GLS) method. Columns 1–6 represent regressions with different independent variables.

**Table 7:** Results of regression with Driscoll-Kraay standard errors (robust)

(a) Pooled least square estimates (POLS) method						
Variable	(1)	(2)	(3)	(4)	(5)	(6)
$lnL{P}_{it}$	0.199^*^ (0.000)	0.120^*^ (0.000)	− 0.005 (0.810)	− 0.048 (0.381)	0.116^*^ (0.000)	− 0.111^*^ (0.000)
$ln{PA}_{it}$	–	0.032^*^ (0.005)	0.032^*^ (0.002)	0.025^*^ (0.001)	0.013^*^ (0.232)	0.057^*^ (0.000)
$ln{RD}_{it}$	–	–	0.237^*^ (0.000)	0.303^*^ (0.000)	0.311^*^ (0.000)	0.097^*^ (0.001)
$ln{ICT}_{it}$	–	–	–	0.123^*^ (0.000)	0.134^*^ (0.000)	0.093^*^ (0.000)
$ln{CS}_{it}$	–	–	–	–	0.043 (0.003)	− 0.052^*^ (0.000)
$ln{EN}_{it}$	–	–	–	–	–	4.667^*^ (0.000)
${\beta }_{0i}$	− 0.142 (0.179)	0.453^*^ (0.037)	1.666^*^ (0.000)	0.764^*^ (0.000)	− 0.602 (0.025)	− 13.226^*^ (0.000)
*F*	605.92	555.49	3325.92	982.22	1281.80	7346.27
Prob. > *F*	0.000	0.000	0.000	0.000	0.000	0.000
*R*-squared	0.075	0.109	0.166	0.221	0.243	0.694
RMSE	0.350	0.344	0.334	0.323	0.319	0.203
Obs	266	266	266	266	266	266
(b) Random-effects (RE) generalized least squares (GLS) method						
$lnL{P}_{it}$	− 0.067 (0.386)	− 0.416^*^ (0.000)	− 0.448^*^ (0.000)	− 0.327 (0.000)	− 0.011 (0.873)	− 0.037 (0.372)
$ln{PA}_{it}$	–	0.260^*^ (0.000)	0.240^*^ (0.000)	0.199^*^ (0.000)	0.216^*^ (0.000)	0.071^*^ (0.004)
$ln{RD}_{it}$	–	–	0.128 (0.291)	0.183 (0.183)	0.258^*^ (0.017)	0.199^*^ (0.014)
$ln{ICT}_{it}$	–	–	–	0.131^*^ (0.000)	0.084^*^ (0.000)	0.022 (0.193)
$ln{CS}_{it}$	–	–	–	–	− 0.230^*^ (0.000)	− 0.216^*^ (0.000)
$ln{EN}_{it}$	–	–	–	–	–	4.638 (0.000)
${\beta }_{0i}$	2.869^*^ (0.000)	4.409^*^ (0.000)	4.838^*^ (0.000)	3.488^*^ (0.000)	3.402^*^ (0.000)	− 11.406^*^ (0.000)
Wald chi^2^	0.79	134.95^*^	333.17^*^	382.38^*^	405.38^*^	7101.91^*^
Prob. > chi^2^	0.374	0.000	0.000	0.000	0.000	0.000
*R*-squared	0.075	0.055	0.066	0.095	0.081	0.392
Obs	266	266	266	266	266	266

The figures in the parentheses () denote probability values. ^*^5% significance level

#### Panel Granger causality method

The Dumitrescu-Hurlin panel Granger causality test for the causality between *Y* and *X* during the period *T* for *N* units. The following heterogeneous model for each unit (*i*) at time *t* is as follows (Dumitrescu and Hurlin 2012):

7 $${Y}_{i,t = {\alpha }_{i}}+\sum_{k=1}^{K}{\gamma }_{i}^{(k)}{Y}_{i,t-k}+\sum_{k=1}^{K}{\beta }_{i}^{(k)}{x}_{i,t-k}+{\varepsilon }_{i,t}$$

where “*K*” symbol denotes optimum lag length and ${\alpha }_{i}$ illustrates that individual effects are constant. In addition, it is accepted that the autoregressive parameter ${{\gamma }_{\dot{\mathrm{I}}}}^{\left(k\right)}$ and the regression coefficient slope ${\beta }_{\dot{\mathrm{I}}}^{(k)}$ can differ between groups. The basic and alternative hypotheses tested using Eq. (7) are as follows:

8 $$\begin{array}{c}\begin{array}{cc}{H}_{0}={\beta }_{i}=0& {\forall }_{i}=1,\dots , N\end{array}\\ \begin{array}{ccc}{H}_{1}={\beta }_{i}=0& {\forall }_{i}=1,\dots , N& 0\le { N}_{1}/N<1\end{array}\\ \begin{array}{cc}{\beta }_{i}\ne 0& {\forall }_{i}={N}_{1},\dots , N\end{array}\end{array}$$

Under the basic hypothesis (*H*_0_), among the variables of all units examined, there is no Granger causality and if otherwise (the alternative hypothesis, *H*_1_), there is a significant relationship. Meanwhile, the other details about the estimation procedure and interpretations are provided in the literature.

## Empirical results and discussion

We first focus on the POLS estimation results in panel (a) (see Table 7). Accordingly, the estimated labor productivity (*lnLP*) parameters (columns 1–2 and 5–6) are statistically significant. Except for column 6, there is a positive relationship between labor productivity and carbon dioxide emissions. In other words, an increase in labor productivity boosts carbon dioxide emissions, thus responsible for more environmental degradation. Interestingly, the studies of Fitzgerald et al. (2018) and Simionescu et al. (2021) are among the rare investigation of the nexus between environmental quality and labor productivity (proxy as working hours). Specifically, these studies corroborate the observation in the current examination because Fitzgerald et al. (2018) found that working hours at state levels in the United States of America spurred carbon emissions during 2007–2013, while Simionescu et al. (2021) noted that working hours caused an increase and decline in GHG emissions in the Old European Union (EU) and new EU member states respectively.

Additionally, it is shown that patent (*lnPA*) parameter estimates are statistically significant and positively impact (lnCO_2_) for five model specifications. The research and development expenditures (*lnRD*) parameter also shows statistically significant and has a positive impact on (*lnCO*_2_) for four model specifications. Information and communication technology (*lnICT*) and energy (*lnEN*) parameter estimates reveal statistically significant and a positive impact on *(lnCO*_2_) for three models and one model specifications, respectively. It is seen that the capital stock (*lnCS*) parameter estimates are statistically significant. But the result in column 5 shows that an increase in capital stock causes a surge in *lnCO*_2_ emission (not statistically significant) while the result in column 6 shows the opposite. Looking at the results in our main equation with all independent variables expressed in column 6, we found that a 1% increase in *lnLP* and *lnCS* reduced carbon dioxide emissions by 0.11% and 0.05%, respectively. In contrast, the result also shows that 1% increase in *lnPA*, *lnRD*, *lnICT*, and *lnEN* parameters augments *lnCO* by 0.05%, 0.097%, 0.093%, and 4.6%. As can be seen, all interpreted parameters are significant according to the t statistic. Comparing these outcomes with the existing literature, it is found that Yang and Liu (2022) and Churchill et al. (2019) established that R&D expenditures mitigate environmental degradation in the Chinese industrial sectors and G-7 economies, respectively, while Garrone and Grilli (2010) noted that public energy R&D failed to exert a statistically significant influence on carbon factor and carbon intensity in selected advanced economies. Additionally, while Adedoyin et al. (2020) and Onifade and Alola (2022) both affirm the desirable roles of R&D and environmental-related innovations in improving environmental quality, Zhou et al. (2019) and Ganda (2019) allay the fear that environmental-related emissions are driven by increasing demand for ICT products and of patent families/researchers, respectively.

Moreover, diagnostically, the *R*-square results in column 6 show that 69.4% of the independent variables used can explain the dependent variables, and other variables can explain the remaining 31.6%. However, the results obtained by the robust pooled least squares method may be biased as it neglects the individual effect, time effect, or both. Therefore, we examine the results of the random-effects (RE) generalized least squares (GLS) method, which provides consistent and efficient parameter estimates in the presence of an individual effect, as we have determined before.

Second, the RE-GLS estimation results are provided in panel (b). Although the coefficients are different, we see that the POLS-based estimation results in panel (a) and the RE-GLS-based estimation results in panel (b) have similar observations, especially for the environmental effects of the transformations in technology. According to panel (b) results, the estimated *lnLP* parameters (columns 2, 3, and 4) are statistically significant and harm *lnCO*_2_. The *lnPA* parameter estimates are statistically significant and indicate a positive impact on *lnCO*_2_ for five model specifications. In comparison, the *lnRD* parameter estimates are statistically significant and reveal a positive impact on *lnCO*_2_ for two model specifications (in columns 4–5). Similarly, the *lnICT* and *lnEN* parameter estimates have a statistically significant and a positive impact on lnCO_2_, whereas *lnEN* parameter estimates are seen to be statistically significant and exert a positive impact on *lnCO*_2_. When the RE-GLS estimation results including all independent variables in column 6 are evaluated, it is observed that a 1% increase in *lnPA*, *lnRD*, and *lnEN* increases *lnCO*_2_ by 0.07%, 0.19%, and 4.6%, respectively. In comparison, a 1% increase in *lnCS* reduces *lnCO*_2_ by 0.21% by a statistically significant degree. In general, the estimations’ results show that technological advances lead to an upsurge in carbon dioxide emissions.

### Panel Granger causality

Table 8 results illustrate the panel causality relationship between variables. According to this result, there are unidirectional causality relationships from *lnPA*, *lnICT*, and *lnCS* to *lnCO*_2_. The results reveal that the parameters that determine technology trigger carbon emissions. Although this observation aligns with the above-highlighted coefficient estimates, it is not desirable to further mention that investments in technology lead to an increase in carbon dioxide emissions, thus contradicting a carbon–neutral program and strategies. These findings disclose the importance of harmonizing the technological advances with the environment.

**Table 8:** Panel Granger causality test results

Direction of causality	Chi-sq	df	Prob
$ln{LP}_{it}\to {lnCO2}_{it}$	4.277	2	0.117
$ln{PA}_{it}\to {lnCO2}_{it}$	8.952	2	0.011^b^
$ln{RD}_{it}\to {lnCO2}_{it}$	1.774	2	0.411
$ln{ICT}_{it}\to {lnCO2}_{it}$	17.672	2	0.000^a^
$ln{CS}_{it}\to {lnCO2}_{it}$	7.850	2	0.019^b^
$ln{EN}_{it}\to {lnCO2}_{it}$	1.859	2	0.394
All	64.686	18	0.000^a^

^a,b,c^Significance at 1%, 5%, and 10% levels, respectively. The test results give the panel Granger causality test results for homogeneous panels

To reach more reliable results in the panel causality test, it is necessary to determine whether the panel is homogeneous or heterogeneous. For this purpose, the Swamy S homogeneity test was used before Dumitrescu-Hurlin’s (2012) panel causality considering the suitability of the Dumitrescu-Hurlin (2012) panel causality for heterogeneous estimation. Although the step-by-step illustration of the approach is not detailed here for lack of space, the result is depicted in Table 9. As seen in Table 9, there is a unidirectional causality relationship between labor productivity (LP) and CO_2_, a bidirectional relationship between patent number variable (PA) and CO_2_. Additionally, there is a unidirectional causality from research and development expenditures and information communication technologies (ICT) to carbon dioxide emissions. Lastly, we have found a bidirectional causality relationship between capital stock and carbon dioxide emissions. Generally, the Dumitrescu-Hurlin Granger causality test reveals that technological developments and investments without eco-sensitive design lead to carbon dioxide emissions. Thus, the study provides insight about the profit-centered production activities of the examined countries.

**Table 9:** Dumitrescu-Hurlin (2012) panel causality test

Null hypothesis:	W-Stat	Zbar-Stat	Prob	Decision
$ln{LP}_{it}\to {lnCO2}_{it}$	4.531	2.712	0.006^a^	Unidirectional causality from $ln{LP}_{it}$ to ${lnCO2}_{it}$
$ln{CO2}_{it}\to {lnLP}_{it}$	3.272	1.111	0.266	
$ln{PA}_{it}\to {lnCO2}_{it}$	3.845	1.840	0.065^c^	Bidirectional causality from $ln{PA}_{it}$ to ${lnCO2}_{it}$
${lnCO2}_{it}\to ln{PA}_{it}$	4.064	2.119	0.034^b^	
$ln{RD}_{it}\to {lnCO2}_{it}$	3.129	0.928	0.353	There are no causality relationships
${lnCO2}_{it}\to ln{RD}_{it}$	3.416	1.293	0.195	
$ln{ICT}_{it}\to {lnCO2}_{it}$	4.102	2.166	0.030^b^	Unidirectional causality from $ln{ICT}_{it}$ to ${lnCO2}_{it}$
${lnCO2}_{it}\to ln{ICT}_{it}$	3.280	1.120	0.262	
$ln{RD}_{it}\to {lnCO2}_{it}$	2.257	− 0.228	0.819	Unidirectional causality from ICT to lnCe
${lnCO2}_{it}\to ln{RD}_{it}$	4.919	3.022	0.002^b^	
$ln{CS}_{it}\to {lnCO2}_{it}$	6.019	4.607	0.000^a^	Bidirectional causality from $ln{CS}_{it}$ to ${lnCO2}_{it}$
${lnCO2}_{it}\to ln{CS}_{it}$	3.904	1.915	0.055^c^	
$ln{EN}_{it}\to {lnCO2}_{it}$	2.719	0.406	0.684	There are no causality relationships
${lnCO2}_{it}\to ln{EN}_{it}$	2.145	− 0.324	0.745	

^a,b,c^Significance at 1%, 5%, and 10% levels, respectively. Swamy S chi^2^(91) = 15,469.39 Prob. > chi^2^ = 0.000. When the probability value of the Swamy S homogeneity test was compared with the 0.05 significance level, it was determined that the null hypothesis was rejected, and this panel had heterogeneous characteristics, not homogeneous. According to this result, we can perform Dumitrescu and Hurlin (2012) causality analysis being suitable for the heterogeneous panel

## Conclusion and policy dimension

Historically, human interaction with nature has revolutionized, thus characterizing several stages of both desirable and somewhat undesirable aspects. The process of transforming nature to generate a product that is (not) necessarily alien to nature is essentially possible with the use of labor tools and sometimes with the various division of labor. Additionally, technological advances and innovations in economic activities are consistently being recalibrated to meet present day challenges. However, the developments above from the aspects of nature, labor participation, technological advances, and innovations are strongly incorporated into the subject of climate change. Thus, this study examined the role of high technology export, labor productivity, patent applications, research and development, and information and communication technology in mitigating environmental degradation for the selected panel of high-tech investing developed countries over the period 2000–2018. According to the Durbin-Hausman test result, there is a significant long-term relationship between explanatory variables and carbon dioxide emission in the panel countries, which aligns with many studies that suggests that technology variables are important environmental factors.

The analysis results show that it is appropriate to use the one-way individual effect model as the final model. The Hausman test determines whether the individual effect affects the independent variable. Accordingly, the investigation found that the random-effects model is valid. Other necessary pre-tests reveal the existence of deviations from the assumption, such as heteroscedasticity, autocorrelation, cross-sectional dependence, and the use of robust estimators like the Driscoll-Kraay standard error robust estimators. Accordingly, the coefficient estimation reveals interesting perspectives. For instance, the indicators of technological progress such as the number of patents, research and development, information and communication technology, and total energy utilization increase carbon dioxide emission. However, there is statistically significant evidence that capital stock mitigates the emission of carbon dioxide, thus promoting environmental quality in the panel of examined countries. The panel Granger causality results also show a causal relationship from the number of patent applications, exports of information and communication technologies, and capital stock to carbon dioxide emissions. However, the current study is limited in scope because it does not provide the environmental performance response to the sectoral advancement in technological innovations. As such, there should be consideration of this limitation in future implementation.

### Policy

Since over a century, humanity has been faced with increasing ecological problems, especially those associated with GHG emissions. Although a lot of success has been achieved, especially in the development of energy technologies and renewable sources, the result of this study suggests the need for a regular review of technological innovation channels to further improve the environmentally compatible technologies across the globe. Specifically, relevant actor should further review the guidelines for patent applications to accommodate more stringent measures or conditions for environmentally disadvantaged inventions. The motives for innovation should be guided and centered on sustainability dimensions and only driven by return on investment and profit mechanism. Moreover, decision-makers could provide more incentives such as patent application subsidy, access to financing, and other measures to encourage more interest in environmentally friendly investments and innovations.

## Data Availability

Data are available upon request from the corresponding author.

## Appendix

Table

**Table 10:** Patent applications by income group, 2010 and 2020

	Number of applications		Resident share (%)		Share of world total (%)		Average growth (%)
Income group	2010	2020	2010	2020	2010	2020	2010–2020
High income	1,393,900	1,552,800	61.8	56.0	69.8	47.4	1.1
Upper middle income	515,500	1,617,100	66.1	86.0	25.8	49.4	12.1
Lower middle income	78,300	104,900	32.8	40.4	3.9	3.2	3.0
Low income	9700	1900	89.7	31.6	0.5	0.1	− 15.0
**World**	**1,997,400**	**3,276,700**	**61.9**	**70.3**	**100.0**	**100.0**	**5.1**

Source: WIPO Statistics Database, September 2021

^10^

## Nomenclature

ARDL: Autoregressive distributed lag
BLUE: Best linear unbiased estimator
CS: Capital stock
CCS: Carbon capture and sequestration
CCS: Carbon capture and storage
CCU: Carbon capture and utilization
CO_2_: Carbon dioxide
CEP: Carbon emissions performance
CSP: Concentrated solar power
DW: Durbin Watson
EKC: Environmental Kuznets curve
EN: Energy
GDP: Gross domestic product
GHG: Greenhouse gas
GLS: Generalized least squares
GMM: Generalized method of moments
ICT: Information and communication technology
IPAT: Impact of population, affluence, and technology
LB: Ljung-Box test
LP: Labor productivity
NGMCPI: Non-radial global Malmquist carbon emission performance index
OECD: Organization for Economic Co-operation and Development
PA: Patent applications of residents
PWT: Penn World Table
POLS: Pooled ordinary least square
QARDL: Quantile autoregressive distributed lag
R&D: Research and development
RE: Random effects
SDGs: Sustainable Development Goals
UNCTAD: United Nations Conference on Trade and Development
UNDP: United Nations Development Program
UTPAT: Utility patent
VIF: Variance inflation factor
WEF: World Economic Forum
WIPO: World Intellectual Property Organization

## Footnotes

1 The selected countries are China, Denmark, Finland, France, Israel, Korea, Hong Kong, Germany, Japan, Netherlands, Singapore, Sweden, United Kingdom, and United States.
